# Measuring the extent of overlaps in protected area designations

**DOI:** 10.1371/journal.pone.0188681

**Published:** 2017-11-27

**Authors:** Marine Deguignet, Andy Arnell, Diego Juffe-Bignoli, Yichuan Shi, Heather Bingham, Brian MacSharry, Naomi Kingston

**Affiliations:** 1 UN Environment World Conservation Monitoring Centre (UNEP-WCMC), Cambridge, United Kingdom; 2 International Union for Nature Conservation (IUCN), Gland, Switzerland; Hawaii Pacific University, UNITED STATES

## Abstract

Over the past decades, a number of national policies and international conventions have been implemented to promote the expansion of the world’s protected area network, leading to a diversification of protected area strategies, types and designations. As a result, many areas are protected by more than one convention, legal instrument, or other effective means which may result in a lack of clarity around the governance and management regimes of particular locations. We assess the degree to which different designations overlap at global, regional and national levels to understand the extent of this phenomenon at different scales. We then compare the distribution and coverage of these multi-designated areas in the terrestrial and marine realms at the global level and among different regions, and we present the percentage of each county’s protected area extent that is under more than one designation. Our findings show that almost a quarter of the world’s protected area network is protected through more than one designation. In fact, we have documented up to eight overlapping designations. These overlaps in protected area designations occur in every region of the world, both in the terrestrial and marine realms, but are more common in the terrestrial realm and in some regions, notably Europe. In the terrestrial realm, the most common overlap is between one national and one international designation. In the marine realm, the most common overlap is between any two national designations. Multi-designations are therefore a widespread phenomenon but its implications are not well understood. This analysis identifies, for the first time, multi-designated areas across all designation types. This is a key step to understand how these areas are managed and governed to then move towards integrated and collaborative approaches that consider the different management and conservation objectives of each designation.

## Introduction

Modern societies are exerting increasing pressures on natural resources, ecosystems and landscapes [1]. Rapid consumption and unsustainable use of natural resources has been connected to environmental degradation, resource scarcity, and a decline in biodiversity [2,3,4]. The role of protected areas as a valuable tool against these pressures on biodiversity, and their related effects on human populations is now well recognized [5,6,7]. Today, protected areas are present in almost every country of the world [8]. The benefits they deliver to society include provision of water, food and medicine, and they also provide important recreational, educational, spiritual and cultural places [5,9,10,11].

Although the preservation of areas for biodiversity conservation is an ancient concept [5,12,13], it was in the 20^th^ century that the contemporary concept of protected areas spread around the world, propelled by different driving forces from one region to another. The expansion of the protected area network worldwide has been partly stimulated by the development of international and national targets, implemented to respond to the unsustainable use of natural resources. Examples include the Aichi Biodiversity Targets of the Convention on Biological Diversity (CBD), and particularly Target 11, which calls for the protection of 17% of the world’s terrestrial area and 10% of its marine area [14].

Over the past decades, the definition of a protected area has evolved to include a range of governance regimes and management styles, including indigenous reserves, game reserves and watershed protected forests, among many others [6]. In this context, governance refers to the processes by which decisions about a protected area are made, and who makes those decisions, e.g. a government agency or a local community [15]. Management refers to the actions that take place as a result of the protected area’s governance. A growing acknowledgment that these connected concepts can vary significantly across sites has led to an expansion of the protected area concept, and in an increasing recognition of a diverse range of sites. These different types of protected area are often referred to using different designations, e.g. National Park or Communal Conservancy. Within a country or administrative division, protected areas sharing the same designation will often have similar management regulations, supporting legislation and conservation objectives. These designations can vary significantly across and within countries, and regions but can be grouped into 3 generic groups: national designations (those that are created by the country under national regulations, for example the Brazilian Reservas Biológicas), regional designations (created by regional processes, for example the Special Areas of Conservation in Europe), and international designations (created by international conventions and agreements such as the World Heritage Convention).

In 1962, around 9,000 sites were reported in the first United Nations List of Protected Areas. By the publication of the fourteenth edition in 2014, this figure had risen to over 209,000 [8]. The growth of the global protected area network over the past few decades, both in numbers and in designation types, reflects countries’ commitment to conserving Earth’s natural habitats. It further shows that protected areas have become a higher priority on most governments’ agendas [10]. At the global level, the development by the International Union for the Conservation of Nature (IUCN) of an internationally-recognised system of protected area management and governance categories [15,16] provides a framework for describing the diversity of protected areas. Furthermore, the creation of international policy processes that designate protected areas, notably the United Nations Educational, Scientific and Cultural Organization (UNESCO) World Heritage Convention, the UNESCO Man and Biosphere programme and the Ramsar Convention on Wetlands, provides further evidence for growing global commitment to the protected area concept [17]. While these designations share the same overall goal of conservation, and are in line with sustainable management objectives, they each have their own specific purpose and management requirements [18]. The World Heritage Convention designates sites that are deemed to be of Outstanding Universal Value [19]. The Man and Biosphere Convention designates reserves that reconcile the conservation of biodiversity with its sustainable use [20]. The Ramsar Convention aims to protect wetlands (which includes a variety of inland and coastal habitats) by promoting national and international cooperation for the conservation and wise use of these areas [21].

One of the effects of the diversification of protected areas is the multiplication of the number of locations, both marine and terrestrial, that are covered by more than one designation (e.g. a National Park that is also a World Heritage Site). This may occur, for example, when a site’s multiple values are recognized under designations specific to each value. This is, for example, the case when a site is protected for its biodiversity, its hydrology and its historical significance by different national or international mechanisms [18]. This multi-level designation of a site may be beneficial if the legal structure means that additional protection is conferred through each designation. However, it may also introduce the risk of conflicting management objectives or governance structures, with potential negative impacts on management, or on local people who are dependent on the area’s resources.

In other cases, overlapping designations may be a by-product of the increasing recognition afforded to non-government protected areas, i.e. those under the governance of indigenous peoples, local communities and private entities [15]. Such overlaps can be identified in the World Database on Protected Areas (WDPA) which is the most comprehensive database on protected areas [22]. These are cases where governments have designated protected areas that overlap with existing traditional or private designations, which have themselves been reported to the WDPA as protected areas. Notably, many countries do not report designations under these governance types to the WDPA [23,24], which means that these types of overlap are likely to be far more common on the ground than is reflected in the database.

Assessing the extent to which overlaps of protected area designations occur is a necessary step to understand how these areas are being managed and governed but to date this has not been undertaken at a global level and across all designation types. This analysis would be fundamental to identify potential duplication of efforts and synergies in a same conservation area and, when necessary, highlight the need to implement an integrated and collaborative management that takes into account the management and conservation objectives of each designation.

Here we assess the extent of the multiple designations phenomenon at three scales (global, regional, and national) and for two ecological realms (terrestrial, and marine). The aim is to understand the extent of overlapping designations, by measuring the number and area of such overlaps.

## Methods

### Data collation and preparation

The April 2016 version of the WDPA was used for this analysis [25]. The analysis uses only polygon data, i.e. protected areas for which a spatial boundary is available in the WDPA. Point data were excluded (i.e. data for which a latitude/longitude location is available, but no boundary), since methods of buffering these points to create artificial boundaries could have generated artefact overlaps. With point data excluded, the dataset used contains 202,528 protected areas (hereafter referred to as ‘protected area layer’). The terrestrial administrative boundaries used in the analysis are taken from the World Vector Shoreline (WVS) dataset. The administrative boundaries in the marine realm were created by combining this dataset with a layer of Exclusive Economic Zones (EEZ) and Areas Beyond National Jurisdiction (ABNJ). For the regional analysis, countries were grouped into the eight regions defined by the UN Environment. A list of countries and territories included in the different regions are provided in S1 Appendix. Detailed information on the data collation and preparation is provided in S2 Appendix.

### Analyses

The spatial analysis was completed in ArcGIS (v.10.3) and using the Arcpy library. The non-spatial analysis was carried out using R (Version 0.98.1103). Detailed information about the processing is provided in S2 Appendix.

Three levels of analysis were performed: global, regional and national. To ensure policy relevance and alignment with global indicators we use the UN Environment (UNEP) regions, a widely used and predefined regional classification of the world [8, 26, 27, 28, 29, 30]. For each region in turn, the protected area layer was clipped by the outline of that region using the administrative layer. Protected areas were filtered based on their country code (‘ISO3’) to include only those associated with that region (see S1 Appendix), thus preventing protected areas on regional borders being included in the wrong region. This filtering was not applied for Areas Beyond National Jurisdiction (ABNJ) and for protected areas in overseas territories. Protected area designations were divided into subsets using the WDPA’s English designation field (‘DESIG ENG’) and the designation type field (‘DESIG_TYPE’) which distinguishes between national, regional and international designations. To identify overlaps between designations, each designation was spatially intersected with the others. These intersections generated new polygons representing areas of overlap. To find the number of overlaps, an ID number was created for each of the resulting polygons based on the coordinates of its centre point (centroid), and the number of designations with the same ID was calculated. The area of each polygon was also calculated. All polygons with an area smaller than or equal to 0.001km^2^ were removed from the analysis. This value represents an empirical threshold aimed at eliminating artefacts caused by the spatial misalignment in protected area borders and administrative borders. This step may not remove all possible artefacts, however those that remain are likely to be small and have a negligible impact on results at the regional and global levels.

Regional statistics were calculated for number of sites, areas of total protected area coverage and areas of overlap–including between different international designations and national designations. Global figures for number of sites, areas of coverage and areas of overlap were derived by aggregating regional statistics.

A national level analysis was carried out using the same methods as above, but instead using country outlines to clip the protected area layer, and filtering to include only protected areas whose country code (‘ISO3’) matched that country. This distinction in the filtering step means that national results do not aggregate to the regional or global levels, however we do provide regional and global results as explained above. Sites listed under regional agreements and conventions were grouped with national designations for the purpose of this study. This is because the application of regional conventions and agreements varies, in a similar way to national designations, between countries and regions. In contrast, international (global) agreements and conventions are more consistently applied and are therefore treated as distinct from national designations in this analysis.

## Results

### Overlaps in terrestrial areas

#### Global scale

**Fig 1:**
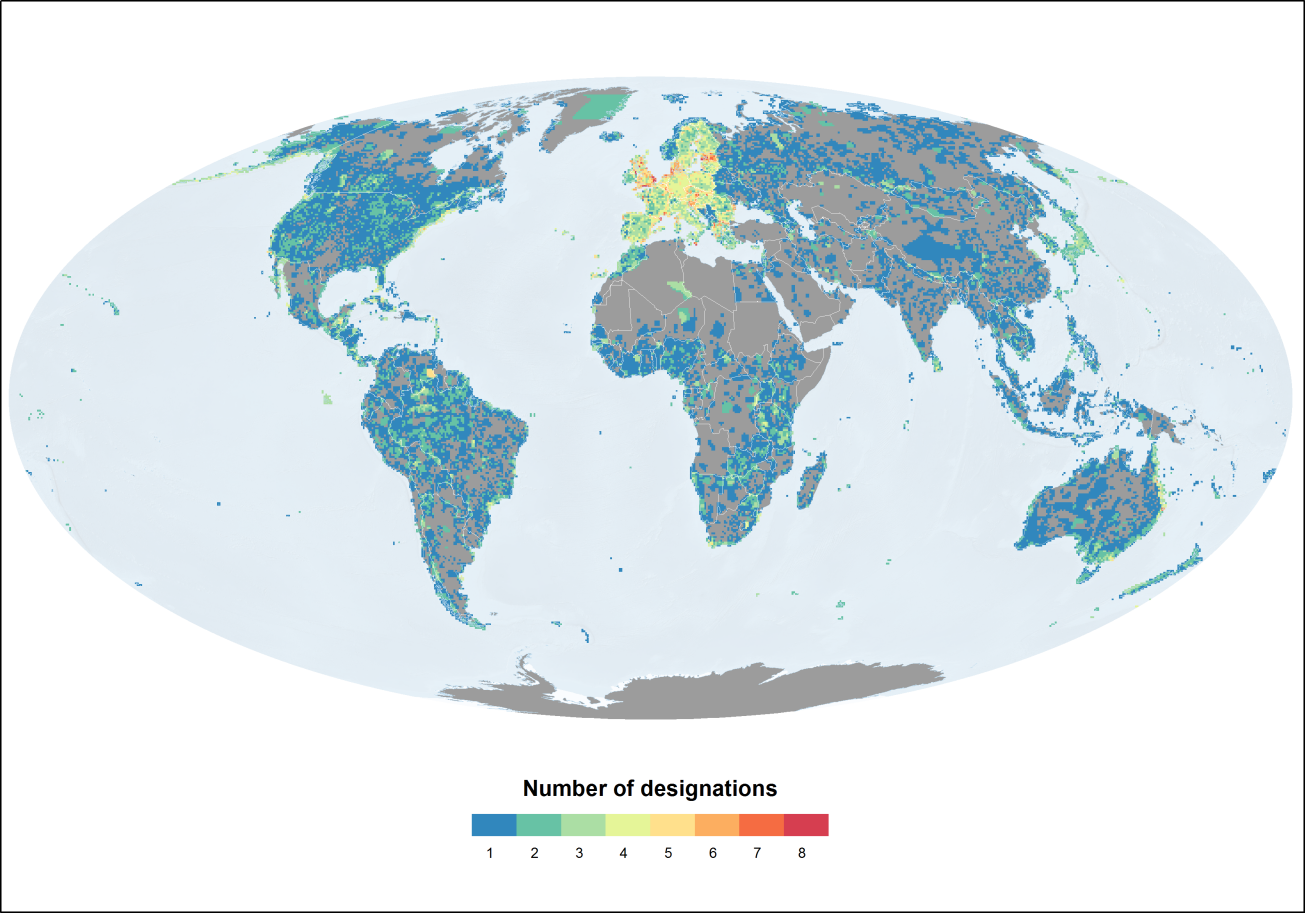
Distribution of the terrestrial protected area network by number of designations. The gradation in colours reflects the increase in the number of overlaps, from one designation (no overlap) (blue) to eight designations (overlap) (red). This includes protected areas designated at the national, regional and international levels.

About a quarter of the global protected area network is protected through two or more designations (overlap) (Fig 1). Although the majority of the network is protected under one type of designation only areas protected through two or more designations occur in every region, most notably in Western Europe. Most areas with only one designation are protected under a national level designation (three quarters of the global protected area network) (Table 1). A relatively small extent is protected through one international designation (about 4% of the global network).

**Table 1:** Global terrestrial network: Percentage of overlap within and between national and international designation types. (WHS: World Heritage Site, MAB: UNESCO Man and Biosphere Reserve). The letter N is used as an abbreviation for national designation. In this table, regional designations are considered as national designations (see section methodology section in S2 Appendix for further details).

		INTERNATIONAL DESIGNATIONS							
									WHS
						MAB	WHS	WHS	MAB
		No. inter-national desig.	Ramsar	MAB	WHS	Ramsar	Ramsar	MAB	Ram-sar
NATIONAL DESIGNA-TIONS	No. national desig.	0	1.536	1.096	0.936	0.041	0.092	0.043	< 0.001
	N1	75.984	1.867	6.798	4.073	0.106	0.472	1.030	0.023
	N2	3.801	0.160	0.129	0.279	0.018	0.011	0.007	< 0.001
	N3	1.010	0.097	0.012	0.047	0.001	0.023	< 0.001	0.001
	N4	0.145	0.027	0.003	0.094	0.001	0.003	< 0.001	< 0.001
	N5	0.022	0.006	< 0.001	< 0.001	< 0.001	< 0.001	0	0
	N6	0.003	< 0.001	0	< 0.001	0	< 0.001	0	0
	N7	< 0.001	< 0.001	0	0	0	0	0	0
	N8	< 0.001	0	0	0	0	0	0	0

**Fig 2:**
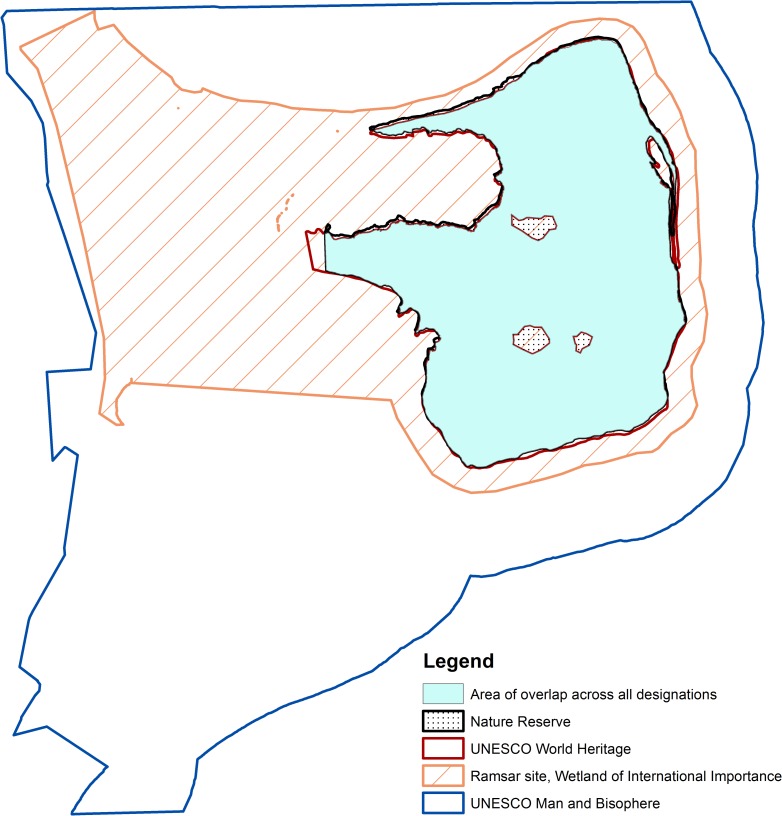
Overlapping protected area designations at Peninsula Valdés, Argentina.

A closer analysis of the portion of the terrestrial network that presents overlaps shows that the protection of a site through a national designation in conjunction with an international designation is the most recurrent type of overlap (Table 1), followed by the protection of a site through two national designations. Although the higher the number of designations covering a site, the lower the area concerned is, the table shows that some areas are covered by five or more national designations (in Europe for example) (S3 Appendix). Other areas are covered by all three international designations as well a national level designation (less than 1% of the global network). This is the case for example for some areas in Latin and South America, including Peninsula Valdés in Argentina (Fig 2) as well as in the Caribbean (S3 Appendix). Areas protected through the highest number of designations (eight) do not include any international designations.

Peninsula Valdés, in Argentina, is protected through a national level designation and under the three international designations. The establishment of the area as a nature reserve in 1983, integrated all previously designated protected areas, with a view to guide responsible tourism development. This same area was inscribed as a UNESCO World Heritage Site in 1999, in recognition of the significant natural habitats and outstanding universal value of some of its species. Ramsar Site of International Importance was designated in 2012 to protect some of the wetlands associated with tidal areas in the Peninsula, which provide nesting and resting sites to numerous migratory sites. In 2012, the area was also designated as a UNESCO Man and Biosphere Reserve combining the protection of the fragile marine and terrestrial ecosystems and the sustainable development of the area. The total area protected by these four designations is of 3,370 km2, which represents over 95% of the size of the nature reserve and the World Heritage Site.

#### Regional scale

Four regions, Africa, Asia and the Pacific, Europe, and Latin America and the Caribbean, contribute to 83% of the global coverage of protected areas (Table 2). The North American region and the Polar region networks each represent between 12% and 5% of the global network, and the West Asia region has the lowest contribution to the global network (less than 1%).

**Table 2:** Key regional statistics on terrestrial protected area (PA) coverage and percentage overlaps for national and/or international designations.

Region	Total land area, km^2^	Contribution of region to global PA network, %	PA coverage, %	Number of max. overlaps*	Total area covered with max. overlaps, km^2^	Total number of PAs
**Africa**	30,048,460	20.04	12.87	5 (4)	0.34 (2442.5)	6,577
**Asia + Pacific**	31,130,830	21.85	13.55	7 (5)	0.39 (109.36)	25,855
**Europe**	27,811,745	16.77	11.64	8	39.02	130,074
**Latin America + Caribbean**	20,541,112	24.05	22.60	6 (5)	0.11 (17713.8)	6,585
**North America**	19,445,662	11.92	11.82	6 (4)	0.24 (126.22)	32,991
**Polar**	16,111,823	4.72	5.66	3	1738.33	34
**West Asia**	3,533,476	0.65	3.53	3 (2)	1.88 (890.61)	133

*If the total area covered by the highest number of designations was less than 10 km^2^, we considered there could not be a high degree of confidence in the results, since they could be from the summing artefacts of the spatial analysis rather than being true overlaps. In these instances, we also reported in parenthesis the second highest number of designations (Number of max. overlaps) and its associated coverage (Total area covered with max. overlaps, km^2^).

Regardless of the total extent of the protected area network at the regional scale, overlaps in designation can be observed in every region. The maximum number of designations protecting an area varies across the regions, from a maximum of two designations providing protection to a site in West Asia or the Polar region to a maximum of eight designations in Europe. The higher number of designations overlapping in Europe is partly due to the additional protection status provided by regional level designations (e.g. Natura 2000 network), which for the purpose of this analysis were considered as national designations (See methods). The regional level designations currently reported to the WDPA are mostly in Europe and predominantly cover marine areas: out of a total of seven regional designations represented in the WDPA, five specifically cover the European region and five are exclusively marine designations. The overlap patterns in marine protected areas were analysed separately and results are presented below.

**Fig 3:**
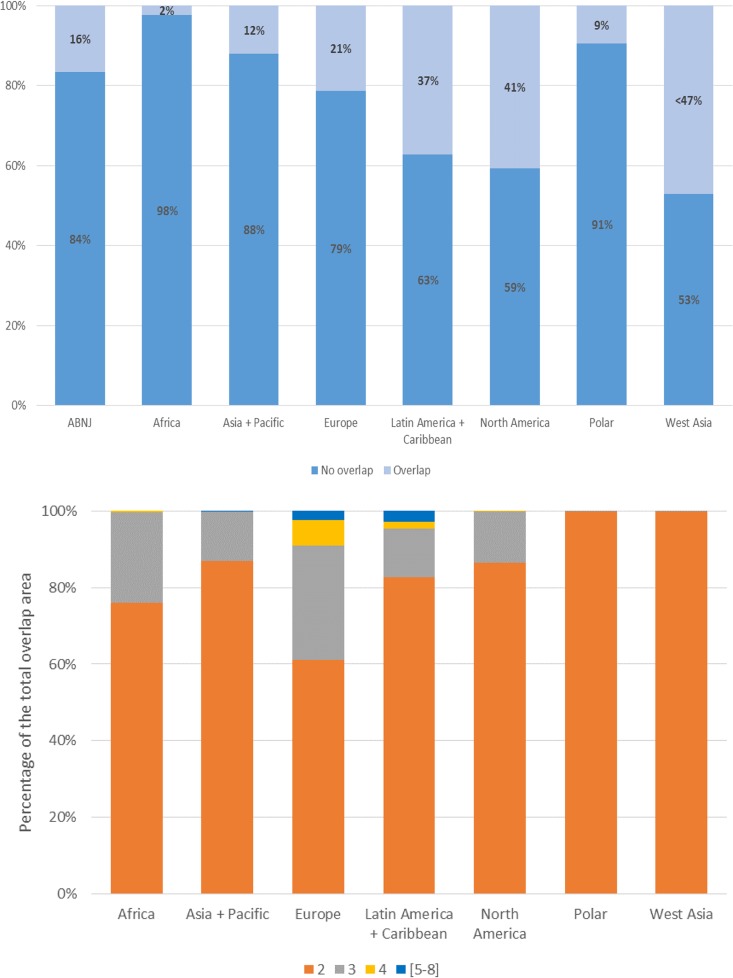
**A: Total terrestrial protected area coverage (in percentage) protected through one designation (no overlap) and through multiple designations (overlap). B: Percentage of total terrestrial overlap area, separated by number of overlaps.** Note: this only refers to the areas of overlap as identified by the light blue portion of the bar charts in Fig 3A.

The total area covered by multiple designations also varies across the regions (Fig 3A). Areas protected by a single designation (no overlap) are common in all regions, varying from 75% of the network in Europe to 86% in Latin America and the Caribbean and to 99% in West Asia. Within the area protected by a single designation, national level designations provide the largest contribution to coverage. By contrast, protection provided by a single international designation is low, ranging from 1% to 7% of the regional networks (S3 Appendix). Only the Polar region presents a different pattern, with most of its area under protection (96%) covered by an overlap consisting of one national and one international designation.

In all regions, the higher the number of designations protecting an area, the smaller the corresponding area becomes (Fig 3B). In Africa for example, with a contribution of 20% to global protected area coverage, 80% of the region’s network is protected by a single designation only, 15% by two designations, 5% by three designations and less than 1% by four or more designations. A similar pattern can be seen in the other regions.

Overlap by two designations is the most common type of overlap in all regions. However, the combination varies across regions. The combination of a national designation and a World Heritage Site is the most recurrent overlap and provides the largest contribution to the total area protected by two designations in Africa, Asia and the Pacific, Europe and North America (S3 Appendix). In the Polar region and West Asia, the combination of a national designation and a UNESCO Man and Biosphere site is the most recurrent overlap, and also covers the greatest area. In Latin America and the Caribbean, overlap of two national designations is the most recurrent model (S3 Appendix).

#### National scale

Tables presenting comprehensive results for every country are provided in S4 Appendix (results for the terrestrial realm) and S5 Appendix (results for the marine realm). Out of 219 countries and territories, 36 countries and territories do not display any overlap, meaning that their entire terrestrial protected area network is protected through single designations. In contrast, eight countries and territories have more than 90% of their terrestrial protected area network covered by more than one designation (overlap). Only Kiribati has its entire terrestrial network protected by overlapping designations.

**Fig 4:**
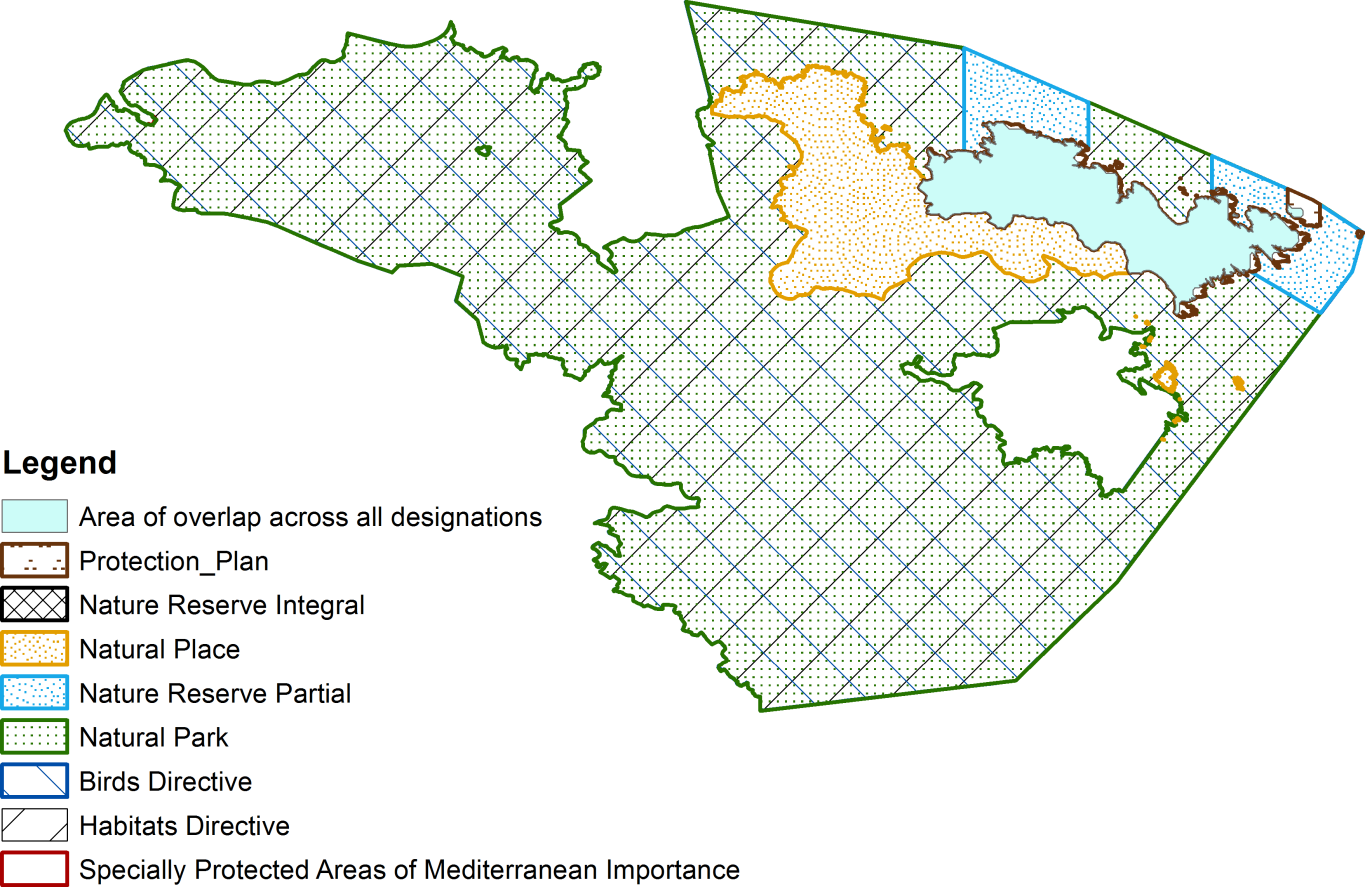
Overlapping protected area designations at Cap de Creus, Spain.

The percentage overlap between international designations only in each country was also calculated: 28 countries and territories have between less than 10% of their terrestrial protected area network covered by multiple international designations. Conversely, 129 countries and territories do not have any areas covered by more than one international designation. Two of these, Guadeloupe and Luxembourg, have more than 90% of their terrestrial protected area network covered by multiple national designations. Cap de Creus in Spain is an example of site protected through eight designations (Fig 4).

According to the WDPA, Cap de Creus, in Spain, is an example of a site protected through eight designations (three of which are regional level designations labelled as national for the purpose of this analysis). The establishment of a protection plan in 1992 provided the foundation for the designation of the area as a Site of Community Importance (Habitats Directive). In 1998, a Nature Reserve and a Natural Place designations were created to encompass the Protection Plan area, and a Natural Park was also created on the area protected through the Habitats Directive designation. In 2001, a Special Protected Area of Mediterranean Importance was created under the Barcelona Convention and an Area of Special Importance was created in 2006 under the Birds Directive. The total area commonly protected by eight designations is of 8.335 km2, which represents over 95% of the size of the nature reserve and protection plan area.

### Overlaps in marine protected areas

#### Global scale

**Fig 5:**
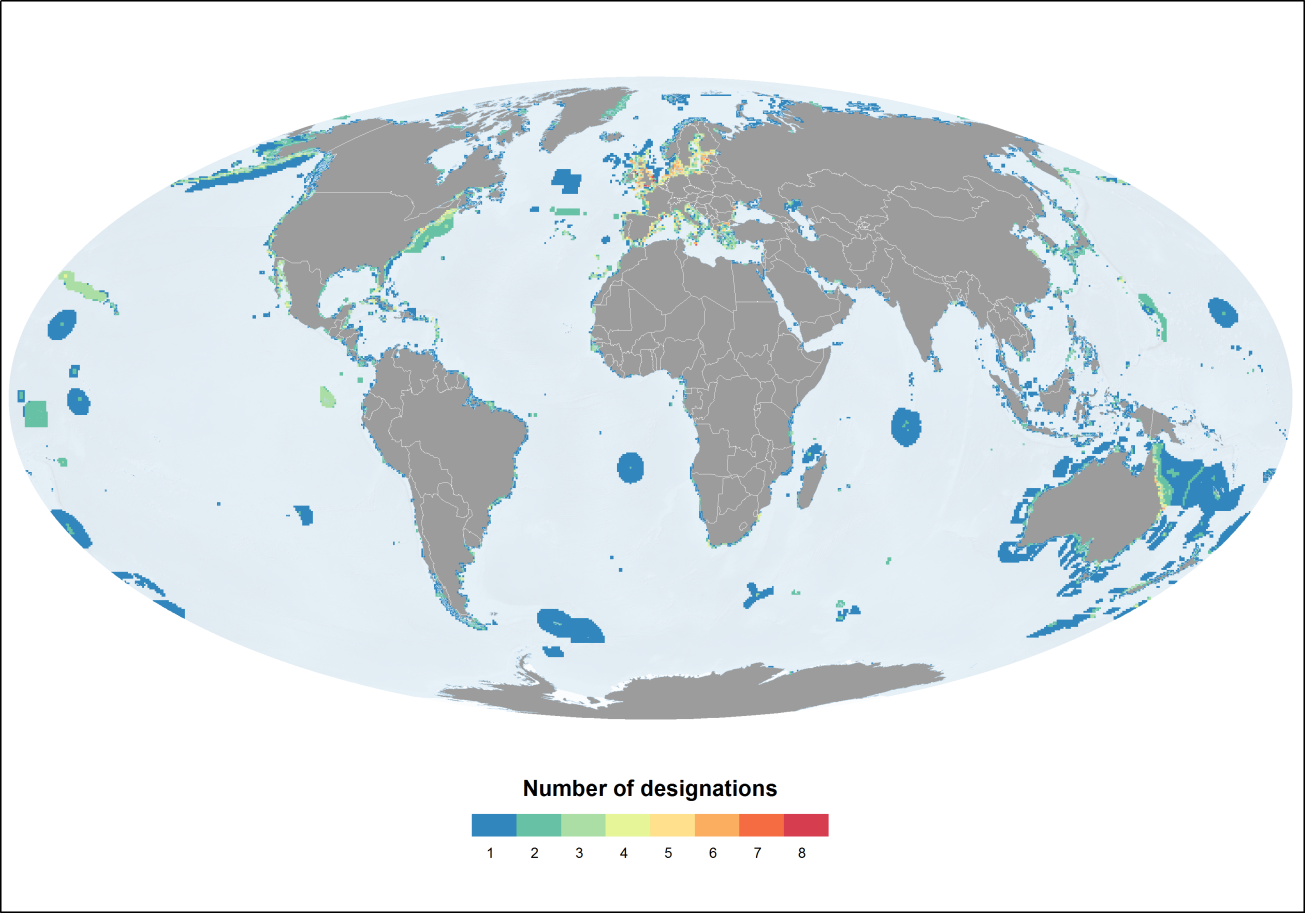
Distribution of the marine protected area network by number of designations. The graduation in colours reflects the increase in the number of overlaps, from one designation (no overlap) (blue) to eight designations (overlap) (red). This includes protected areas designated at the national, regional and international levels.

Under a fifth of the global marine network is protected by two or more designations (Fig 5). The configuration of overlaps in the marine realm is very similar to that in the terrestrial realm: it is Europe that marine areas are protected through the highest number of designations (Table 3). The analysis shows that marine areas closer to the shoreline tend to be protected by a higher number of designations than more remote marine areas.

**Table 3:** Key regional statistics on marine protected areas coverage and percentage overlaps for national and/or international designations.

Region	Total marine area, km^2^	Contribution of region to global PA network, %	PA coverage, %	Number of max. overlaps*	Total area covered with max. overlaps, km^2^	Total number of PAs
**ABNJ**	221,134,569	4.32	0.28	2	103181.95	60
**Africa**	15,561,254	5.61	5.21	5 (4)	0.05 (757.96)	492
**Asia + Pacific**	63,312,155	59.21	13.53	7 (6)	0.45 (20.80)	4,353
**Europe**	17,542,705	5.64	4.65	8	13.77	11,592
**Latin America + Caribbean**	21,371,698	4.02	2.72	5 (4)	0.01 (2036.59)	1,404
**North America**	14,301,943	16.80	16.99	6 (5)	0.05 (11.96)	2,687
**Polar**	6,218,074	8.68	20.18	3	456.61	32
**West Asia**	1,443,769	0.06	0.56	3 (2)	0.09 (3812.36)	48

*If the total area covered by the highest number of designations was less than 10 km^2^, we considered there could not be a high degree of confidence in the results, since they could be from the summing artefacts of the spatial analysis. In these instances, we also reported in parenthesis, the second highest number of designations (Number of max. overlaps) and its associated coverage (Total area with max. overlaps, km^2^).

#### Regional scale

**Fig 6:**
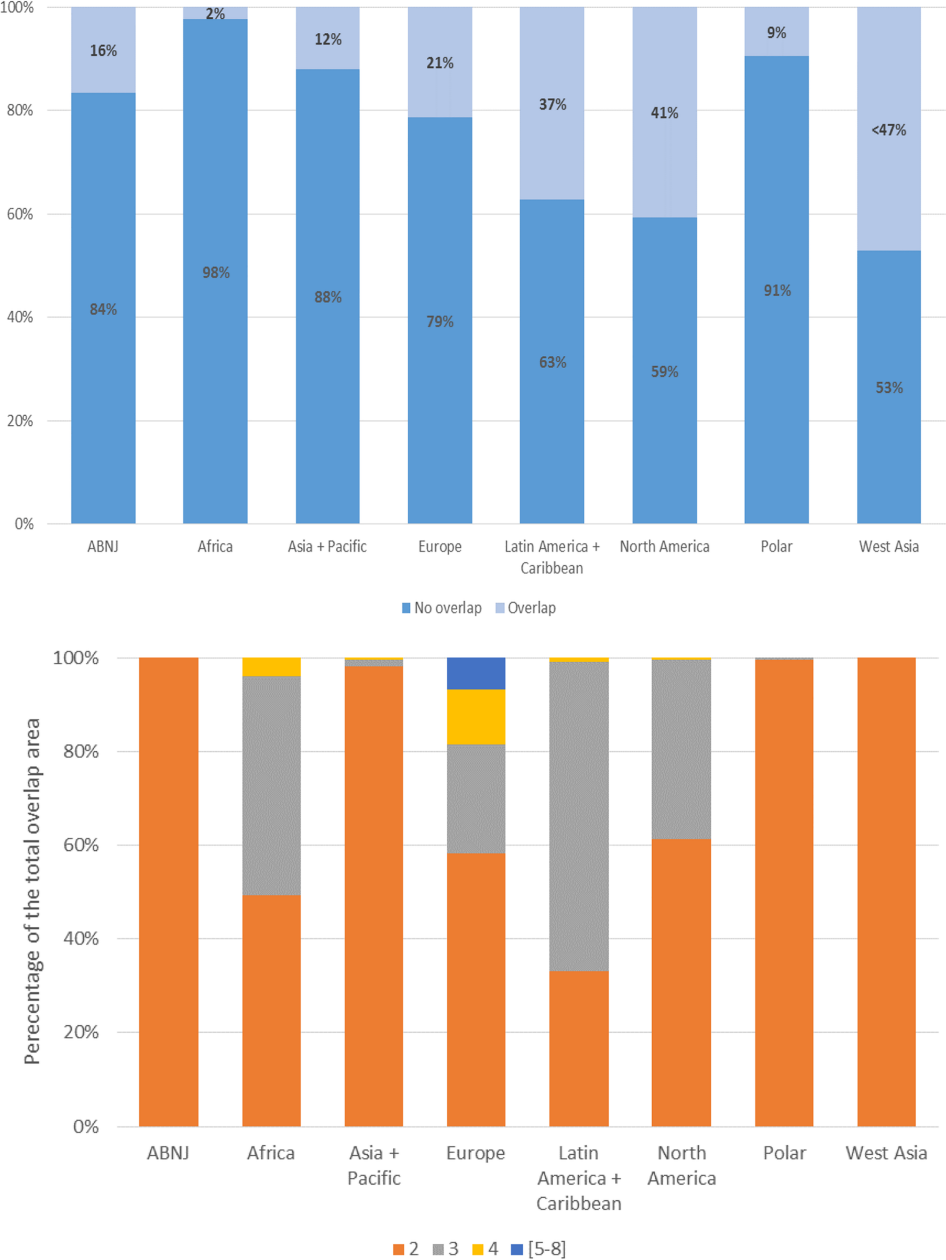
**A: Total marine protected area coverage (in percentage) protected through one designation (no overlap) and through multiple designations. B: Percentage of total marine overlap area, separated by number of overlaps.** Note: this only refers to the areas of overlap as identified by the light blue portion of the bar charts in Fig 6A.

Overlaps in marine protected area designations are present in every region (Fig 6A), to different extents (Fig 6B).

In Europe, Africa and Latin America and the Caribbean, the existence of regional conventions partially explains overlaps in marine protected area designations. Such regional designations include marine protected areas designated under the OSPAR Convention, established to protect and conserve the North-East Atlantic and its resources, and the Helsinki Convention on the protection of the marine environment in the Baltic Sea area in Europe. In Northern Africa, regional level marine protected areas are established through the Barcelona Convention and its associated SPAMI sites (Specially Protected Areas of Mediterranean Importance), covering the Mediterranean area. In Latin America and the Caribbean, the Cartagena Convention designates regional marine protected areas referred to as specially protected areas.

As in the terrestrial realm, the use of international designations and national designations to protect areas differs between regions. In contrast to Europe and North America, where overlaps are mostly among national designations, in Africa and the Polar region the most common overlaps are between a national designation and an international MAB site. In Asia and the Pacific, and Latin America and the Caribbean, overlaps between a national designation and a World Heritage Site cover the greatest area. In West Asia, overlaps of a national designation and a Ramsar site are the most recurrent.

#### National scale

Out of 175 countries and territories (including the ABNJ), 59 countries and territories do not show any overlap, meaning that their entire marine protected area network is protected through single designations (full results are provided in S5 Appendix). Conversely, 18 countries and territories have more than 90% of their marine protected area network covered by more than one designation (overlap), two of which have their entire marine network protected by multiple designations only.

The percentage overlap between international designations only reveals that 14 countries and territories have less than 14% of their marine protected area network covered by multiple international designations. A further 79 countries and territories have overlaps consisting of multiple national designations only. Four of these (Estonia, Lithuania, Malta and Poland) report more than 90% of their marine protected area network as covered by multiple national designations.

## Discussion

Our analyses show that sites designated under a single designation provide the largest contribution to the global network of protected areas, for both the marine and terrestrial realms. However, a consequence of the expansion and diversification of the global protected area network is the increasing number of locations that are being protected by more than one designation.

A quarter of the global terrestrial network, and just under a fifth of the global marine network, is protected by two or more designations. These overlaps in protected area designations occur in every region of the world. The amount of overlap reported in each region does not appear to be correlated with the total area of its protected area network. Europe, for example, has the highest maximum number of overlaps between designations. The region also reports the highest number of sites in total. However, it does not have the highest protected area coverage. One reason for this is that Europe is a very populated region and, although large numbers of protected areas are designated, they are typically small. In comparison, Africa reports a much smaller total number of protected areas, but this network protects a far larger land area than is protected in Europe.

While the areas that overlap include different combinations of designations, the overlap between one (or several) national designations with at least one international designation is most recurrent. The addition of an international designation to a national-level protected area can be valuable as it accentuates the importance of the area for biodiversity conservation at the global level. This can be of benefit by indirectly promoting the development of the wider region through creating broader income opportunities [18].

Multiple national and/or international designations may be of benefit if this means that the multiple values of an area are recognized and protected through several complementary mechanisms. It can also provide several advantages, for example increasing the resilience of these areas to external pressures, or raising the visibility and prestige of these areas, which can in turn help promote increased tourism [18]. However, it is important to ensure coordinated management efforts among the different management authorities involved.

In addition to potential advantages, there may be challenges associated with the governance and management of areas protected through more than one designation. In particular, challenges may arise when areas are managed simultaneously under the governance of different national and/or international agencies, or of governments and non-government actors. This could lead to conflicts if the objectives and management requirements of the different designations are not compatible. In addition, a lack of coordination between the different authorities in charge can result in the inefficient use of staff and funding (for example through multiplication of reporting efforts), or in competition over financial support [18, 31]. A lack of coordination between the different actors in charge of managing the different designations might result in ineffective management, with the potential result that the site becomes a ‘paper park’ [31]. For example, in an area with up to 8 different designations such as in Cap de Creus in Spain, which may have different management needs under each designation, it is important that the area is managed under the stricter of these designations.

In order to improve the management of such an area, it is essential that governance authorities develop a shared understanding of the different objectives and management requirements of the different designations, and strengthen collaboration and coordination efforts.

Regional differences in the scale of overlaps can be explained by variations in the history, perception of, and approach to protected areas, which varies from one region to another. In Africa, the current protected area network has historical links to colonial times, and is composed of a small number of national designations, complemented by international designations, designed to protect the continent’s rich ecosystems and biodiversity [32]. On the other hand, the high amount of protected areas overlaps in Europe, and Western and Central Europe more particularly, is explained by the region’s political efforts to prioritize environmental issues and to foster cooperation among the countries [32]. The network is characterized by a wide and expanding range of national-level designations, as well as regional-level designations. One notable example of this is the Natura 2000 network adopted by the EU governments in 1992 [33]. The aim of the network is to protect habitats and species across Europe listed in the Habitats Directive and Birds Directive. This resulted in the creation of two protected area designations: Sites of Community Interest and Special areas of Conservation which, in addition to existing national designations, explains the high amount of overlap.

Finally, as explained above, this study does not assess the implications of multi-designated areas for on the ground conservation and resource management. Understanding these would be the next area of research after having identified where these places are. It is important that the management authorities of each overlapping designation establish a collaborative and integrate management and governance of the area. This would help, for example, to ensure conservation objectives are being responded to and emerging threats to wildlife are being effectively addressed. Coordinated efforts could help to reduce poaching in African protected areas, where hunting of elephants for the illegal ivory trade has reduced elephant populations at a rapid rate over the past two decades [34, 35]. For example, forest elephants in Central Africa declined by ca. 62% between 2002 and 2011 [36]. If staff from the different designations joined their efforts and coordination, this could lead to improved effectiveness and reduction of costs.

Such an effort could not be achieved without improved resource management. Integrated and collaborative management could also involve bringing together the revenues generated by tourism in the different areas through the different designations. For example, in the case of Peninsula Valdés in Argentina, revenues generated from the World Heritage label, UNESCO Man and Biosphere Reserve, Ramsar and national park labels would increase the area’s budget available. In addition, areas under multiple designations such as Valdes in Argentina or Cap de Creus in Spain have to engage to different reporting requirements over different time periods, whether they are reporting to international, regional or national processes. These could perhaps be integrated under a common process that would save important time and resources.

## Conclusion

The present analysis quantified the overlaps in protected area designations at a global, regional and national level using the best global protected areas dataset available. Three key messages can be drawn from this exercise. Firstly, overlap in protected area designations occurs in every region of the world, both in the marine and terrestrial realms, but is more prominent in the terrestrial realm and in some regions more than others. Secondly, in all regions, drivers for establishing protected areas under national or international designations are different, resulting in regional networks that have different characteristics, and different degrees of overlap. Finally, the higher amount of overlap reported in Europe is strongly influenced by regional designations.

This work furthers our understanding of the extent and number of overlaps in protected areas designations at different scales. The results constitute an important consideration for management and governance authorities, and for actors involved in the designation of protected areas. This study complements the important work done by Schaaf and Rodrigues (2016) on the extent to which international designations overlap. However, the present study does not address the important question of whether the multi-designation phenomenon is beneficial or detrimental for nature conservation and whether it enhances or hinders effective management of protected areas. Building on this, further work could focus on the effectiveness of management in areas protected through multiple designations, and comparing locations with a high number of designations with locations protected through only one designation to evaluate whether the additional protection conferred to a site is more effective in conserving biodiversity.

## Supporting information

S1 Appendix List of countries and territories included in the UN Environment regional divisions used in the analysis. (DOCX)

S2 Appendix Data preparation and processing. (DOCX)

S3 Appendix Percentage of the terrestrial regional protected area network covered by one to eight national designations and one to three international designations in the different UN Environment regions. (XLSX)

S4 Appendix National level country overlap statistics in the terrestrial realm. (XLSX)

S5 Appendix National level country overlap statistics in the marine realm. (XLSX)
