# Supplementary material for: Measuring the extent of overlaps in protected area designations
Source: PLoS One. 2017 Nov 27;12(11):e0188681. doi: 10.1371/journal.pone.0188681 (PMC5703568; doi:10.1371/journal.pone.0188681)
Supplement: S1 Appendix — (DOCX) [file pone.0188681.s001.docx]

## S1 Appendix: List of countries and territories included in the UN Environment regional divisions used in the analysis.

The regional groupings used in this study follow those defined by the UN Environment (<https://www.grid.unep.ch/geo/region.htm>).

| **Region** | **Countries and territories included** |
| --- | --- |
| **ABNJ** | ABNJ |
| **Africa** | Angola, Terres Australes et Antarctiques Françaises, Burundi, Benin, Burkina Faso, Botswana, Central African Republic, Cote d’Ivoire, Cameroon, Democratic Republic of the Congo, Republic of Congo, Comoros, Cape Verde, Djibouti, Algeria, Egypt, Eritrea, Western Sahara, Ethiopia, Gabon, Ghana, Guinea, Gambia, Guinea Bissau, Equatorial Guinea, Kenya, Liberia, Libya, Lesotho, Morocco, Madagascar, Mali, Mozambique, Mauritania, Mauritius, Malawi, Mayotte, Namibia, Niger, Nigeria, Reunion, Rwanda, Sudan, Senegal, Saint Helena, Sierra Leone, Somalia, South Sudan, Sao Tome Principe, Swaziland, Seychelles, Chad, Togo, Tunisia, United Republic of Tanzania, Uganda, South Africa, Zambia, Zimbabwe. |
| **Asia and the Pacific** | Afghanistan, American Samoa, Australia, Bangladesh, Brunei Darussalam, Bhutan, Cocos (Keeling) Islands, China, Cook Islands, Christmas Island, Fiji, Micronesia (Federated States of), Guam, Hong Kong, Indonesia, India, British Indian Ocean Territory, Iran (Islamic Republic of), Japan, Cambodia, Kiribati, Republic of Korea, Lao People’s Democratic Republic, Sri Lanka, Macao, Maldives, Marshall Islands, Myanmar, Mongolia, Commonwealth of the Northern Mariana Islands, Malaysia, New Caledonia, Norfolk Island, Niue, Nepal, Nauru, New Zealand, Pakistan, Pitcairn Island, Philippines, Palau, Papua New Guinea, Democratic People’s Republic of Korea, French Polynesia, Singapore, Solomon Islands, Thailand, Tokelau, Timor-Leste, Tonga, Tuvalu, Taiwan (Province of), United States Minor Outlying Islands (except Navassa), Viet Nam, Vanuatu, Wallis and Futuna, Samoa. |
| **Europe** | Albania, Andorra, Armenia, Austria, Azerbaijan, Belgium, Bulgaria, Bosnia and Herzegovina, Belarus, Switzerland, Cyprus, Czech Republic, Germany, Denmark, Spain, Estonia, Finland, France, Faroe Islands, United Kingdom, Georgia, Guernsey, Gibraltar, Greece, Croatia, Hungary, Isle of Man, Ireland, Iceland, Israel, Italy, Jersey, Kazakhstan, Kyrgyzstan, Liechtenstein, Lithuania, Luxembourg, Latvia, Monaco, Republic of Moldova, The former Yugoslav Republic of Macedonia, Malta, Montenegro, Netherlands, Norway, Poland, Portugal, Romania, Russian Federation, Svalbard and Jan Mayen Islands, San Marino, Serbia, Slovakia, Slovenia, Sweden, Tajikistan, Turkmenistan, Turkey, Ukraine, Uzbekistan, Holy See. |
| **Latin America and the Caribbean** | Aruba, Anguilla, Argentina, Antigua and Barbuda, Bonaire Sint Eustatius and Saba, Bahamas, Saint Barthelemy, Belize, Bermuda, Bolivia (Plurinational State of), Brazil, Barbados, Chile, Colombia, Clipperton Islands, Costa Rica, Cuba, Curacao, Cayman Islands, Dominica, Dominican Republic, Ecuador, Falkland Islands (Malvinas), Guadeloupe, Grenada, Guatemala, French Guiana, Guyana, Honduras, Haiti, Jamaica, Saint Kitts and Nevis, Saint Lucia, Saint Martin, Mexico, Montserrat, Martinique, Nicaragua, Panama, Peru, Puerto Rico, Paraguay, El Salvador, Suriname, Sint Marteen, Turks and Caicos Islands, Trinidad and Tobago, Navassa Island, Uruguay, Saint Vincent and the Grenadines, Venezuela (Bolivarian Republic of), British Virgin Islands, United States Virgin Islands. |
| **North America** | Canada, Saint Pierre and Miquelon, United States of America. |
| **Polar** | Antarctic, Terres Australes et Antarctiques Francaises, Bouvet Island, Greenland, Heard Island and McDonald Islands, South Georgia and the South Sandwich Islands. |
| **West Asia** | United Arab Emirates, Bahrain, Iraq, Jordan, Kuwait, Lebanon, Oman, State of Palestine, Qatar, Saudi Arabia, Syrian Arab Republic, Yemen. |
