# Supplementary material for: Measuring the extent of overlaps in protected area designations
Source: PLoS One. 2017 Nov 27;12(11):e0188681. doi: 10.1371/journal.pone.0188681 (PMC5703568; doi:10.1371/journal.pone.0188681)
Supplement: S2 Appendix — (DOCX) [file pone.0188681.s002.docx]

## S2 Appendix: Data preparation and processing.

### Data preparation

The 2016 April version of the World Database on Protected Areas (WDPA) was used for this analysis (UNEP-WCMC and IUCN, 2016). Protected areas represented as point records (i.e. single latitude/longitude locations) were excluded from the analysis because buffering the points to the protected areas reported area would have produced inaccurate spatial overlaps between sites. Consequently, some sites designated under two of the three international conventions considered in this analysis were excluded because they are represented as point records in the WDPA. The excluded records account for 57% of the UNESCO Man and Biosphere (MAB) dataset (339 sites) and 42% of the Ramsar sites dataset (914 sites). Statistics on international designations are therefore calculated from a subset of the MAB and Ramsar datasets. The World Heritage dataset, for which every site is reported with a polygon boundary, is considered complete. The final polygon layer, hereafter referred to as ‘protected area layer’ used for this analysis contained a total of 202,528 protected areas.

The World Vector Shoreline (WVS) was used as an administrative boundaries layer. The WVS dataset represents national borders for the terrestrial realm (World Vector Shoreline, 3^rd^ edition, National Geospatial-Intelligence Agency). This dataset was combined with a layer of Exclusive Economic Zones (EEZ) and Areas Beyond National Jurisdiction (ABNJ) for the marine realm. Areas falling into disputed territories were removed from the analysis. This layer is hereafter referred to as the ‘administrative boundaries layer’.

For the regional analysis, regional associations corresponding to the UN Environment regions were assigned to the protected area layer and the administrative boundaries layer based on a list of countries in each region (<http://www.grid.unep.ch/geo/region.htm>). Countries were thereby categorized in to eight UN Environment regions. A list of countries and territories included in the different UN Environment regions is provided in S1 Appendix.

From an administrative and management perspective, national governments treat sites designated under regional agreements in the same way as national designations, and regional designations are not applied consistently across countries. For this reason, the seven regional designations were treated as national level designations. For the terrestrial analysis this only affected results in Europe, whereas in the marine analysis this affected Europe, Latin America and the Caribbean (LAC), Africa (Mediterranean sub-region) and Polar regions.

To prepare the protected area layer, transboundary sites (i.e. protected areas with multiple ISO3 codes) were extracted then intersected with the administrative boundaries layer. The English designation field was cleaned (protected areas that appeared to share the same designation except for minor inconsistencies were grouped together, for example ‘National Park’ and ‘National park’). The layer’s geometries were then checked and repaired using ArcGIS tools.

All spatial datasets were projected into an equal area projection (World Mollweide) relevant for global analysis.

### Data processing

Python scripts, utilising the ArcPy package, were used to run the tools for the following processing steps. A list of regions common to both the administrative layer and protected area was created based on the region field in each layer (see Data Preparation and Processing). The list was used to loop through and select polygon features from both the administrative layer and the protected areas layer where the region matched. The Intersection tool was applied to clip the selected protected areas by the selected region boundaries. The Union tool was then used on this clipped layer to intersect different overlapping designations with each other. This produced a layer with a number of new polygons, and where any overlapping polygons would share the same shape and size. The Multipart to singlepart tool was then applied to create a single row per polygon, thus allowing small polygon ‘slithers’ to be highlighted accurately. The Add Field Attributes tool was used to create an area field (km^2^), and all polygons with areas smaller than 0.001 km^2^ were removed. The same tool was then used to add X and Y coordinate fields for the centre (centroid) of each polygon. These coordinate values were concatenated (joined) to a new ID field. Each ID was only shared by overlapping polygons with the same size and shape enabling the number of overlaps to be counted. The attribute table was then exported for the region in question. These steps were repeated to create text files for all regions.

The resulting spatial layers were appended to produce a global layer. Polygons with duplicate IDs were removed and this ‘flattened’ layer was used in subsequent steps for creating Figure 1 (see below).

The statistical software R was then used to create summaries of the data. All designations in the designation type (“DESIG_TYPE”) field listed as “Regional” were recoded to “National” (see Methods section for explanation of this grouping). As we only required distinctions between types of international designations (i.e., World Heritage Site, Ramsar sites and UNESCO-Man and Biosphere Reserves), all national and regional designation names were recoded as “National” in the English designation field (“DESIG_ENG”). The ID column (containing the centroid coordinates) was then used to count the number of overlapping designations. For each of the different regions/countries the total area for the different overlap configurations (e.g., two national designations with one World Heritage Site) was calculated. The resulting table was exported as a text file for manipulation using pivot tables in Excel to produce the final set of tables and graphs. Further summary statistics for Table 1 included calculating the areas of each region in the administrative layer and counting the numbers of protected areas using the WDPA identification field, ”WDPA_ID”.

Global level statistics (Table 1) were created from aggregating regional level results.

The global map (Figure 1) was created by appending a table of designation counts for each polygon and joining it to the corresponding polygon on the flattened layer (see above) based on the ID field. A spatial join was made between this layer and an equal area grid (50km resolution) produced using the Fishnet tool in ArcGIS. The maximum number of overlaps in each grid cell was calculated to provide a visual representation of the broad distribution of designation overlap at the global scale.

Results for the national scale were produced using the same methods as the regional scale, but using the country code (“ISO3”) to select protected areas from the protected area layer and countries from the administrative layer.
